# Clinical, Endoscopic, and Histopathologic Gastrointestinal Disease in an American Cohort With Behçet's Disease

**DOI:** 10.14309/ctg.0000000000000591

**Published:** 2023-04-27

**Authors:** Bryan F. Curtin, Kareen L. Hill, Sumona Bhattacharya, Astin Powers, Aradhana Venkatesan, Preet Bagi, Elizabeth Joyal, Meghna Alimchandani, Raphaela Goldbach-Mansky, Peter Grayson, Martha Quezado, Cailin Sibley, Theo Heller

**Affiliations:** 1 Digestive Disease Branch, National Institute of Diabetes and Digestive and Kidney Diseases, National Institutes of Health, Bethesda, Maryland, USA;; 2 Translational Hepatology Section, Liver Disease Branch, National Institute of Diabetes and Digestive and Kidney Diseases, National Institutes of Health, Bethesda, Maryland, USA;; 3 Laboratory of Pathology, National Cancer Institute, National Institutes of Health, Bethesda, Maryland, USA;; 4 Radiology and Imaging Sciences Department, Clinical Center, National Institutes of Health, Bethesda, Maryland, USA;; 5 Systemic Autoimmunity Branch, National Institute of Arthritis and Musculoskeletal and Skin Diseases, National Institutes of Health, Bethesda, Maryland, USA;; 6 Translational Autoinflammatory Diseases AU2 Section, National Institute of Arthritis andMusculoskeletal and Skin Diseases and National Institute of Allergy and Infectious Diseases, Bethesda, Maryland, USA.

**Keywords:** Behçet's disease, gastrointestinal disease, endoscopy, vascular congestion

## Abstract

**INTRODUCTION:**

Behçet's disease (BD) is a chronic systemic vasculitis characterized by oral and genital ulcers, uveitis, and skin lesions. Patients with BD may develop gastrointestinal (GI) disease; however, characterization of GI disease in American cohorts is lacking. In this article, we present clinical, endoscopic, and histopathologic GI findings in an American cohort of patients with BD.

**METHODS:**

Patients with established BD were evaluated prospectively at the National Institutes of Health. Demographic and clinical data were collected including BD manifestations and GI symptoms. Endoscopy with histopathologic sampling was performed for both clinical and research indications with written consent.

**RESULTS:**

Eighty-three patients were evaluated. The majority were female (83.1%) and white (75.9%). Mean age was 36 ± 14.8 years. GI symptoms were reported in 75% of cohort with nearly half of reporting abdominal pain (48.2%) and significant numbers reporting acid reflux, diarrhea, and nausea/vomiting. Esophagogastroduodenoscopy was performed in 37 patients; erythema and ulcers were the most common found abnormalities. Colonoscopy was performed in 32 patients with abnormalities including polyps, erythema, and ulcers. Endoscopy was normal in 27% of esophagogastroduodenoscopies and 47% of colonoscopies. Vascular congestion was demonstrated on the majority of random biopsies throughout the GI tract. Inflammation was not highly prevalent on random biopsies except in the stomach. Wireless capsule endoscopy was performed on 18 patients; ulcers and strictures were the most common abnormalities.

**DISCUSSION:**

GI symptoms were common in this cohort of American patients with BD. Endoscopic examination was often normal; however, histopathologic examination demonstrated vascular congestion throughout the GI tract.

## INTRODUCTION

Behçet's disease (BD) is a chronic systemic vasculitis characterized by recurrent painful oral and genital ulcers, uveitis or retinal vasculitis, skin lesions, and various other systemic manifestations (1,2). Prevalence of BD in Eastern Asian and Mediterranean regions ranges from 8 to 420 cases per 100,000 (3), whereas in the United States, prevalence is estimated to be lower at 0.38 cases per 100,000 (1). BD has a female predilection, and in an American cohort, peak incidence was found to be in the third decade of life for women and fifth decade of life for men (1,4).

BD is diagnosed based on an internationally established set of criteria comprising the most common manifestations of the disease (2). Although gastrointestinal (GI) disease is not included in this criteria, in our center's experience, patients with BD have commonly reported GI symptoms. A widely variable prevalence of GI symptoms has been reported in BD cohorts at rates ranging from 15% to 92%, including symptoms such as abdominal pain, diarrhea, constipation, and acid reflux (5–9). Intestinal BD is a recognized subcategorization of BD in which patients display predominantly GI symptoms and have documented presence of GI ulcers on endoscopy or surgical pathology (10).

Endoscopic and surgical findings described in BD cohorts are characterized by ulceration throughout the upper and lower GI tracts, particularly in the terminal ileum, ileocecal valve, and cecal regions (5–9,11–13). Ulcers have been described as ranging from subcentimeter aphthous lesions to large ulcers several centimeters in diameter (5,7). Complications from GI disease including ulcer disease have been reported to include weight loss, GI bleeding, obstruction, fistulae, perforation causing peritonitis and sepsis, and death (5,6,8,9,12–16). However, the published research on GI disease in BD is focused on Mediterranean and Asian populations (5–9,11–14,17,18). It is known the frequency of GI involvement in BD varies by country making it important to evaluate an American cohort. It has been shown there is a lower frequency of GI involvement in patients in Turkey, India, and Saudi Arabia but a high frequency in the United Kingdom (19). In this article, we present clinical, endoscopic, and histopathologic GI findings in an American cohort of patients with BD seen at the National Institutes of Health.

## METHODS

Patients were prospectively evaluated at the National Institutes of Health Clinical Center in Bethesda, Maryland, between 2007 and 2013 under an institutional review board–approved protocol (NCT02974595), which studies patients with autoinflammatory diseases including BD. All patients provided written informed consent to participate in this study.

Demographics including sex, race, and age were recorded. The clinical diagnosis of BD was confirmed using ICBD criteria, which designates 2 points each for ocular lesions, oral aphthosis, and genital aphthosis. It also designates a point each for central nervous system involvement or vascular manifestations. Patients who scored 4 or greater were classified as having BD. Current GI symptoms were recorded for each patient at the time of evaluation. Medications used by the patient for control of their disease were also noted. Laboratory studies, which included C-reactive protein, erythrocyte sedimentation rate, white blood cell count, hemoglobin, mean corpuscular volume, platelet count, albumin, and ferritin, were collected.

Initially, endoscopies (esophagogastroduodenoscopy [EGD] and/or colonoscopy) were performed only if clinically indicated, and only abnormality-directed biopsies were obtained. These indications included abdominal pain, diarrhea, nausea, vomiting, hematochezia, early satiety, dysphagia, constipation, and reflux. However, partway through the study, the protocol was amended to allow for performance of endoscopies in asymptomatic patients as well as to obtain random biopsies from the gastric antrum, gastric body, duodenum, terminal ileum, ascending colon, transverse colon, descending colon, sigmoid colon, and rectum. This amendment was made, given the high prevalence of endoscopic and histopathologic abnormalities found in previously evaluated patients with BD. All patients provided consent to undergo endoscopy with biopsies. In addition, wireless capsule endoscopy (WCE) was performed if clinically indicated after obtaining consent.

Pathologic specimens were assessed by an expert GI pathologist (M.Q.) who specifically evaluated these for the presence of vascular congestion and inflammation, as well as any other histopathologic abnormalities.

## RESULTS

A total of 83 patients were evaluated. Demographics are presented in Table 1. A significant majority were female (83.1%) and white (75.9%). The mean age of patients was 36 ± 14.8 years. All patients lived in the United States at the time of evaluation.

**Table 1.** Demographic information of the cohort

Characteristic	No. of patients (%)
Sex	
Male	14 (16.9)
Female	69 (83.1)
Race	
White	63 (75.9)
African American/black	7 (8.4)
Asian	6 (7.2)
Other/unknown	7 (8.4)
Age, yr, mean (SD)	36 ± 14.8

Prevalence of BD manifestations is presented in Table 2. Most patients reported oral ulcerations (88%), and a large proportion also reported genital ulcerations (69.9%). Other manifestations included skin lesions (63.9%), arthralgias (47.0%), and uveitis (30.1%). Pathergy had not been tested in all patients, but documented positive tests were reported in 9.6%.

**Table 2.** Prevalence of Behçet's disease–related manifestations and gastrointestinal symptoms in the cohort

Symptom/manifestation	No. of patients (%)
Behçet's disease–related manifestations	
Oral ulcers	73 (88.0)
Genital ulcers	58 (69.9)
Skin lesions	53 (63.9)
Arthralgias	39 (47.0)
Uveitis	25 (30.1)
Positive pathergy test	8 (9.6)
Gastrointestinal symptom	
Abdominal pain	40 (48.2)
Acid reflux	21 (25.3)
Diarrhea	19 (22.9)
Nausea/vomiting	17 (20.5)
Constipation	15 (18.1)
Hematochezia	12 (14.5)
Dysphagia	10 (12.0)
Weight loss	9 (10.8)

GI symptoms were highly prevalent in our cohort (presented in Table 2) with 75% reporting any GI symptom and abdominal pain reported in nearly half (48.2%). Other commonly reported symptoms included acid reflux (25.3%), diarrhea (22.9%), and nausea/vomiting (20.5%). Hematochezia (14.5%), dysphagia (12.0%), and weight loss (10.8%) were also seen in a significant minority of patients.

Medications at the time of evaluation are presented in Table 3. The most commonly used medications were corticosteroids (42.2%) and colchicine (37.3%). There was also a high prevalence of proton pump inhibitor usage (28.9%). Other agents included azathioprine (20.5%), nonsteroidal anti-inflammatory drugs (NSAIDs) (20.5%), and anti–tumor necrosis factor antagonists (14.5%). Other agents were less commonly used.

**Table 3.** Medication usage in the cohort

Therapy	No. of patients (%)
Corticosteroids	35 (42.2)
Colchicine	31 (37.3)
Proton pump inhibitors	24 (28.9)
Azathioprine	17 (20.5)
Nonsteroid anti-inflammatory drugs	17 (20.5)
Anti–tumor necrosis factor antagonists	12 (14.5)
Mycophenolate mofetil	6 (7.2)
Sulfasalazine	4 (4.8)
Cyclosporine	2 (2.4)
Hydroxychloroquine	1 (1.2)
Vedolizumab	1 (1.2)

Mean laboratory parameters are presented in Table 4. Notably, C-reactive protein was elevated at 6.2 ± 18.1 mg/L. The remainder of the mean laboratory parameters were relatively unremarkable.

**Table 4.** Laboratory findings in the cohort

Laboratory parameter	Normal range	Mean ± SD
C-reactive protein, mg/L	<3.0	6.2 ± 18.1
Erythrocyte sedimentation rate, mm/hr	<25	13.63 ± 12.43
White blood cell count, K/μL	4.23–9.07	7.12 ± 3.34
Hemoglobin, g/dL		
Males	13.7–17.5	13.85 ± 8.90
Females	11.2–15.7	
Mean corpuscular volume, fL	79.4–94.8	87.8 ± 12.39
Platelet count, K/μL	173–369	255.04 ± 61.10
Albumin, g/dL	3.5–5.2	3.89 ± 0.42
Ferritin, μg/L	13–150	61.43 ± 61.74

A total of 37 EGDs and 32 colonoscopies were performed. The most common indication for EGD was abdominal pain, whereas diarrhea was the most common indication for colonoscopy. Endoscopic findings are presented in Table 5. The most common abnormality seen during EGD was gastric erythema (29.7%) followed by duodenal erythema (24.3%). Of those patients with gastric erythema, 1% (n = 1) were on NSAIDs, and 54.5% (n = 6) were on steroids. Of the patients with duodenal erythema, 11.1% (n = 1) were on NSAIDs, and 33.3% (n = 3) were on steroids. Ulcers were seen in 21.6% (n = 8) of cases. Twenty-five percent (n = 2) of those patients were on NSAIDs, and 25% (n = 2) were on steroids. Four patients on NSAIDs who underwent EGD did not have any upper GI tract ulcerations. Less common findings included gastric polyps (13.5%) and esophagitis (10.8%). Four patients (10.8%) exhibited endoscopic signs of possible esophageal dysmotility. The most common findings on colonoscopy were colon polyps (21.9%) and erythema (21.9%). Only 2 (6.3%) patients had ulcers in the colon. Twenty-seven percent of EGDs and 47% of colonoscopies were endoscopically normal.

**Table 5.** Endoscopic findings in the cohort

Endoscopic finding	No. of patients (%)
Esophagogastroduodenoscopy (n = 37)	
Gastric erythema	11 (29.7)
Normal	10 (27)
Duodenal erythema	9 (24.3)
Ulcer(s)	8 (21.6)
Gastric polyp(s)	5 (13.5)
Irregular Z-line	5 (13.5)
Esophageal dysmotility	4 (10.8)
Esophagitis	4 (10.8)
Colonoscopy (n = 32)	
Normal	15 (47)
Colon polyp(s)	7 (21.9)
Erythema	7 (21.9)
Hemorrhoids	5 (15.6)
Ulcer(s)	2 (6.3)
Wireless capsule endoscopy (n = 16)	
Normal	7 (43.8)
Ulcer(s)	5 (31.2)
Stricture	2 (12.5)
Arteriovenous malformation	1 (6.3)
Lymphangiectasias	1 (6.3)

**Figure 1.**
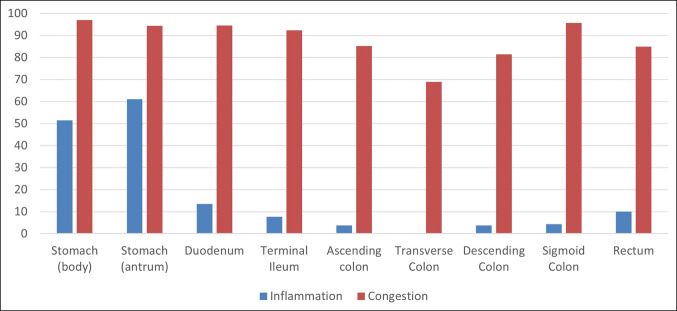
Prevalence of vascular inflammation and vascular congestion seen on histopathologic analysis in different areas of the gastrointestinal tract.

**Figure 2.**
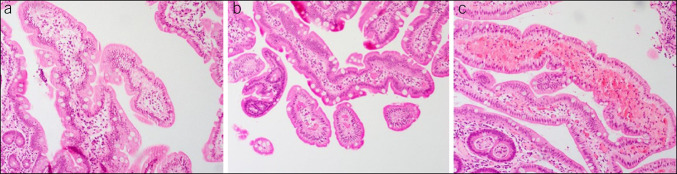
Histopathologic specimens taken from the duodenum in patients with Behçet's disease demonstrating various degrees of vascular congestion. Based on the number of vessels that appeared congested on low power screening, severity of vascular congestion was scored as a 0–1+ (**a**), 2+ (**b**), and 3+ (**c**).

Vascular congestion was highly prevalent throughout the GI tract on histopathologic analysis (Figure 1). Inflammation was not as common but generally more prevalent in the upper GI tract as compared to the lower GI tract. Of the 33 biopsies taken from the gastric body, all samples except 1 demonstrated vascular congestion (97%), whereas inflammation was seen in 52% of biopsies from this area. Ninety-four percent and 95% of biopsies in the gastric antrum and duodenum, respectively, demonstrated vascular congestion (Figure 2). Conversely, 61% and 14% of biopsies demonstrated inflammation in these same areas.

Similar to the upper GI tract, most biopsies demonstrated vascular congestion in the terminal ileum and throughout the colon. Ninety-two percent of terminal ileum biopsies demonstrated vascular congestion, whereas only 8% demonstrated inflammation. Lymphoid aggregates were seen in 15% of terminal ileum biopsies. Inflammation was not as frequent with the highest frequency in the rectum (15% of biopsies). The only other common histopathologic finding in this portion of the GI tract was lymphoid aggregates, which were present at rates of 10%–18% throughout the colon.

WCE was performed in 18 patients (21.7% of the cohort) for clinical indications including hematochezia, imaging suggestive of enteritis, or otherwise unexplained abdominal symptoms. Two capsules were unusable because of gastric retention and technical malfunction. Of the remaining 16 WCEs, 9 showed abnormalities (56.2%) and 7 were normal (43.8%) (Table 5). The most common abnormality was small bowel ulceration (31.2%), which usually appeared in the jejunum or ileum. Small bowel strictures were seen in 12.5%, although these did not obstruct passage of the capsule. Other notable findings included duodenal arteriovenous malformations and lymphangiectasias in the jejunum. Of the 18 total patients who underwent WCE, 16 (88.9%) also underwent EGD, and 14 (77.8%) also underwent colonoscopy. Seven of the 9 patients (77.8%) with abnormal capsule findings also had abnormal EGD findings. Four of the 9 patients (44.4%) with an abnormal capsule had abnormal colonoscopy findings.

## DISCUSSION

In this article, we have presented clinical, endoscopic, and histopathologic GI findings in an American cohort of patients with BD. We found that GI symptoms were present in most patients, including nearly half of our cohort reporting abdominal pain and significant numbers reporting acid reflux, diarrhea, and nausea/vomiting. Endoscopic abnormalities were less common; however, vascular congestion was highly prevalent throughout the GI tract on segmental biopsies.

A proposed algorithm for the diagnosis of intestinal BD based on a cohort of Korean patients with BD takes into account the appearance and location of GI ulcers as well as presence of absence of systemic BD manifestations (10). This algorithm was based on the presence of ileocolonic ulcers. In addition, consensus statements from the Japanese Inflammatory Bowel Disease Research Group recommend diagnosis of intestinal BD if there is an oval-shaped ulcer in the terminal ileum or ulcerations in the small bowel or large bowel with other clinical manifestations of BD (20). Our cohort did not have a high prevalence of lower GI tract ulceration. Prevalence of ulcers throughout the GI tract in our cohort ranged from 6.3% to 31.2% with higher frequencies seen on EGD and WCE than on colonoscopy. Therefore, many of our patients would not meet criteria for intestinal BD by these definitions, although all had met criteria for general BD based on their systematic manifestations of the disease. Ulcers can also be precipitated by NSAID use. In our cohort of the 8 patients with ulcer found on EGD, only 2 of them were using NSAIDs, and 4 patients who were taking NSAIDs did not have ulcers on EGD.

The most common GI symptom in our cohort was abdominal pain, which was present in 48.2% of patients. Abdominal pain in other cohorts has been reported at ranges from 22% to 92% (5–7,9). Generally, the abdominal pain reported by our patients was chronic and mild-moderate in severity. We also noted that 22.9% of our patients reported diarrhea; other cohorts have reported prevalence of diarrhea between 11% and 48% (5–7,9). We also had significant numbers of patients with acid reflux, nausea/vomiting, constipation, and weight loss, which are not reported in any significant numbers in other cohorts, if reported at all (5–8).

We found 10 patients (12%) with dysphagia, 4 patients (10.8%) with endoscopic evidence of esophageal dysmotility, and 4 patients (10.8%) with esophagitis on EGD. Esophageal involvement in BD is not well characterized; however, in 1 cohort of 23 patients with BD, 19% reported upper GI symptoms, 5% had esophagitis on EGD, and 35% (corresponding to 7 patients) of those who underwent esophageal manometry had abnormal findings including decreased lower esophageal pressure, repetitive contractions, and low contraction amplitude; however, only 3 of these patients were symptomatic (11). No patients in our cohort underwent manometry; however, this is a potential area for future study. Conversely, in another study on esophageal disease in BD, dysphagia was reported in only 3.1% of patients (8). Other studies reporting GI symptoms in patients with BD have not reported dysphagia in their cohorts (5–7).

Histopathologic analysis in our cohort was significant for vascular congestion, a nonspecific finding, which, nonetheless, was prevalent at high rates throughout the GI tract. Vascular congestion has not been previously reported in BD cohorts. However, none of our specimens demonstrated vasculitis with no evidence of fibrinoid necrosis or inflammation with wall damage. Previous analysis of surgically resected intestinal specimens has demonstrated ulcers with reactive lymphoid hyperplasia at the ulcer base as well as nonspecific inflammation (necrotic angiitis, thrombophlebitis, and perivenular lymphocyte infiltration) and vasculitis in the submucosa with only 1 report of endoscopic specimen with vasculitis (9,16,21). Our inability to demonstrate vasculitis on endoscopic biopsies may be due to endoscopically obtained biopsies, which only reflect superficial layers of the GI tract, and vasculitis may only involve deeper layers of the GI tract if present. It may also be due to NSAID and corticosteroid use in a significant percent of our patient population. However, superficial GI tract biopsies may still be useful, such as to exclude other diseases that present with similar symptoms and mucosal ulcerations, e.g., Crohn's disease and tuberculosis (10). Furthermore, given our findings, nontargeted biopsies may be considered in patients with BD because vascular congestion is neither a common histologic feature of other diseases nor is seen in healthy patients, and therefore, this may be suggestive of BD. Vascular congestion was seen across all patients without relationship to medication history.

On presentation to our center, we noted a wide variety of medications including a significant proportion of patients on systemic steroids, which is a suboptimal therapy, given adverse events associated with long-term use (22). Optimal medical management of BD differs based on the specific manifestations in each patient and may include colchicine, azathioprine, NSAIDs, and anti–tumor necrosis factor antagonists (23,24). Management of GI disease may include 5-aminosalicylates, thalidomide, and biologic agents such as infliximab and adalimumab, as well as endoscopic and surgical management of complications (23,24).

Limitations of our study include lack of follow-up because we typically evaluated patients for just 1 visit. Our patient population was also predominantly white; therefore, our findings may lack generalizability to other races even within American populations. We also noted a high amount of systemic steroid usage within our cohort, which may affect endoscopy or biopsy results by decreasing inflammatory findings. Conversely, NSAID use in our cohort may have contributed to findings such as ulceration and erythema. In addition the medication history was limited to therapy being used at the time of endoscopy and does not include previous therapies. NSAIDs and corticosteroids might influence EGD findings if they had been concomitantly used; however, previous use would most likely not have an effect on EGD findings.

In conclusion, GI manifestations were commonly seen in our cohort of American patients with BD with the majority reporting GI symptoms and almost half reporting abdominal pain. Endoscopic evaluation with EGD, WCE, and colonoscopy was often normal; however, abnormal lesions included erythema and ulcers. Random biopsies obtained throughout the GI tract demonstrated a high prevalence of vascular congestion, which is not prevalent in other diseases or healthy patients. These findings suggest that patients with BD should be evaluated for GI disease and that random biopsies should be considered during endoscopy because vascular congestion may be suggestive of BD. This can lead to transitioning away from corticosteroids to therapies targeted to BD.

## CONFLICTS OF INTEREST

**Guarantor of the article:** Theo Heller, MD.

**Specific author contributions:** B.F.C.: data collection, data analysis, and writing. K.L.H.: data analysis and writing. S.B.: data collection, data analysis, and writing. A.V.: data collection and data analysis. P.B.: data collection, data analysis, and writing. E.J.: nurse coordinator and data collection. M.A.: data collection and data analysis. R.G.-M.: funding acquisition, data collection, and data analysis. P.G.: data collection and data analysis. M.Q.: data collection and data analysis. C.S.: data collection, data analysis, supervision, and conceptualization. T.H.: conceptualization, writing, supervision, data collection, and data analysis.

**Financial support:** Funding for this study was provided by the National Institute of Arthritis and Musculoskeletal and Skin Diseases, National Institute of Allergy and Infectious Diseases, National Institute of Diabetes and Digestive and Kidney Diseases, and National Cancer Institute.

**Potential competing interests:** None to report.

**Study Highlights**

**WHAT IS KNOWN**

✓ Behçet's disease (BD) is a systemic vasculitis. Although not a part of the diagnostic criteria, patients may develop gastrointestinal (GI) disease.
✓ Characterization of GI disease in American patients with BD is not well described.

**WHAT IS NEW HERE**

✓ GI symptoms were commonly reported in our cohort of American patients with BD.
✓ Endoscopic findings were less severe in our cohort than has been described in other populations.
✓ Histopathologic findings were significant for vascular congestion, which was highly prevalent throughout the GI tract. This has not previously been described in BD.
